# Serum autoantibodyome reveals that healthy individuals share common autoantibodies.

**DOI:** 10.1016/j.celrep.2022.110873

**Published:** 2022-05-31

**Authors:** Mahasish Shome, Yunro Chung, Ramani Chavan, Jin G. Park, Ji Qiu, Joshua LaBaer

**Affiliations:** 1 Virginia G. Piper Center for Personalized Diagnostics, Biodesign Institute, Arizona State University, Tempe, AZ, USA; 2 College of Health Solutions, Arizona State University, Phoenix, AZ, USA; 3 Lead contact

## Abstract

Autoantibodies are a hallmark of both autoimmune disease and cancer, but they also occur in healthy individuals. Here, we perform a meta-analysis of nine datasets and focus on the common autoantibodies shared by healthy individuals. We report 77 common autoantibodies based on the protein microarray data obtained from probing 182 healthy individual sera on 7,653 human proteins and an additional 90 healthy individual sera on 1,666 human proteins. There is no gender bias; however, the number of autoantibodies increase with age, plateauing around adolescence. We use a bioinformatics pipeline to determine possible molecular-mimicry peptides that can contribute to the elicitation of these common autoantibodies. There is enrichment of intrinsic properties of proteins like hydrophilicity, basicity, aromaticity, and flexibility for common autoantigens. Subcellular localization and tissue-expression analysis reveal that several common autoantigens are sequestered from the circulating autoantibodies.

## In brief

Shome et al. performed a meta-analysis to discover the common autoantibodies found in healthy individuals. These common autoantibodies appear and increase during youth and plateau at adolescence. Bioinformatics techniques demonstrate the potential role of molecular mimicry in their production as well as several common intrinsic biochemical properties.

## Graphical Abstract

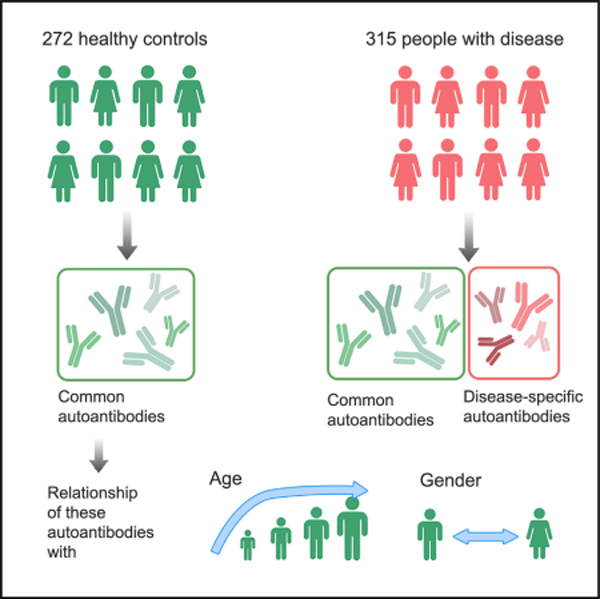

## INTRODUCTION

Autoantibodies have been reported in individuals with autoimmune disease and cancer. They were believed to be absent in healthy individuals due to the immune tolerance mechanism (Nemazee, 2017); however, some have been found frequently in healthy individuals (Nagele et al., 2013), which we call common autoantibodies. These common autoantibodies can confound the search for disease-linked autoantibodies, and their documentation will simplify the identification of autoantibodies specific to certain diseases. Indeed, only a small fraction of the autoantibodies reported in the literature have been validated in independent cohorts (Wang et al., 2016a), suggesting that the classification performance for many reported autoantibodies requires further investigation.

A comprehensive documentation of common autoantibodies will facilitate the elucidation of the complex immunology underlying their elicitation. One class of common autoantibodies is referred to as natural antibodies (NAbs). Unlike adaptive antibodies, NAbs are synthesized by B1 lymphocytes (bearing CD20^+^CD27^+^CD43^+^CD70^−^) and marginal-zone B cells (Griffin et al., 2011; Palma et al., 2018) and do not undergo affinity maturation by antigen stimulation or extensive somatic mutation (Coutinho et al., 1995). Another class of common autoantibodies may arise from cross-reactive antibodies to infectious agent proteins when the similarity in foreign and self peptides may activate self-reactive T or B cells. It has been experimentally demonstrated that patients with either measles virus or herpes simplex virus type 1 produce antibodies against a viral phosphoprotein that cross-react with an intermediate filament protein of human cells (Fujinami et al., 1983). Additionally, transgenic mice infected with lymphocytic choriomeningitis virus (LCMV) may develop chronic inflammation in the central nervous system (CNS) due to epitopes shared between LCMV proteins and CNS antigens (Evans et al., 1996). Several bioinformatics techniques have been developed to discover potential mimicry candidates (Doxey and McConkey, 2013; Ludin et al., 2011; Venigalla et al., 2020).

The immunogenicity of a protein can be attributed to its intrinsic properties and extrinsic responses by the host (Berzofsky, 1985). Biochemical and structural properties like flexibility, hydrophilicity, and beta turns can promote antigenicity, while hydrophobicity, alpha helices, and beta sheets can suppress antigenicity. That these common autoantibodies do not cause evident autoimmune disease is intriguing. The presence of autoantibodies in serum reflects leakiness of central and/or peripheral tolerance mechanisms (Ludwig et al., 2017). However, their presence does not guarantee a causal role in autoimmune-disease development. For autoantibody-induced pathology, the autoantibody needs to bind to the autoantigen to form an immune complex (Suurmond and Diamond, 2015). Sequestration of the autoantigen from autoantibodies can inhibit the autoantibody-induced pathology. In this report, we have performed a meta-analysis of autoantibodyome data from 9 different case-control biomarker studies to identify common immunoglobulin G (IgG) autoantibodies in healthy individuals (Table S1).

## RESULTS

### Identity and prevalence of common autoantibodies

Autoantibody profiles for 272 healthy subjects from 9 case-control studies were compiled (Table S1). There were more females than males, 195 versus 67, because several studies focused on female-specific diseases such as breast and ovarian cancers. These studies were diverse in terms of subject ages, ranging from infancy to adulthood, with most above 50 years old. Antibodies against 8,282 unique human proteins were studied; however, the number of proteins studied for each subject varied by study (Figure S1). To minimize the effect of study heterogeneity, sample-size-based weighted prevalence was calculated as the sum of individual prevalence of antibody in each study multiplied by the sample size of the study. For the healthy subjects, 77 autoantibodies occurred frequently and had a weighted prevalence between 10% and 47% (Table S2). These were termed as common autoantibodies. Antibodies against STMN4, ODF2, RBPJ, AMY2A, EPCAM, and ZNF688 showed the highest prevalence (Table S2).

**Figure 1.**
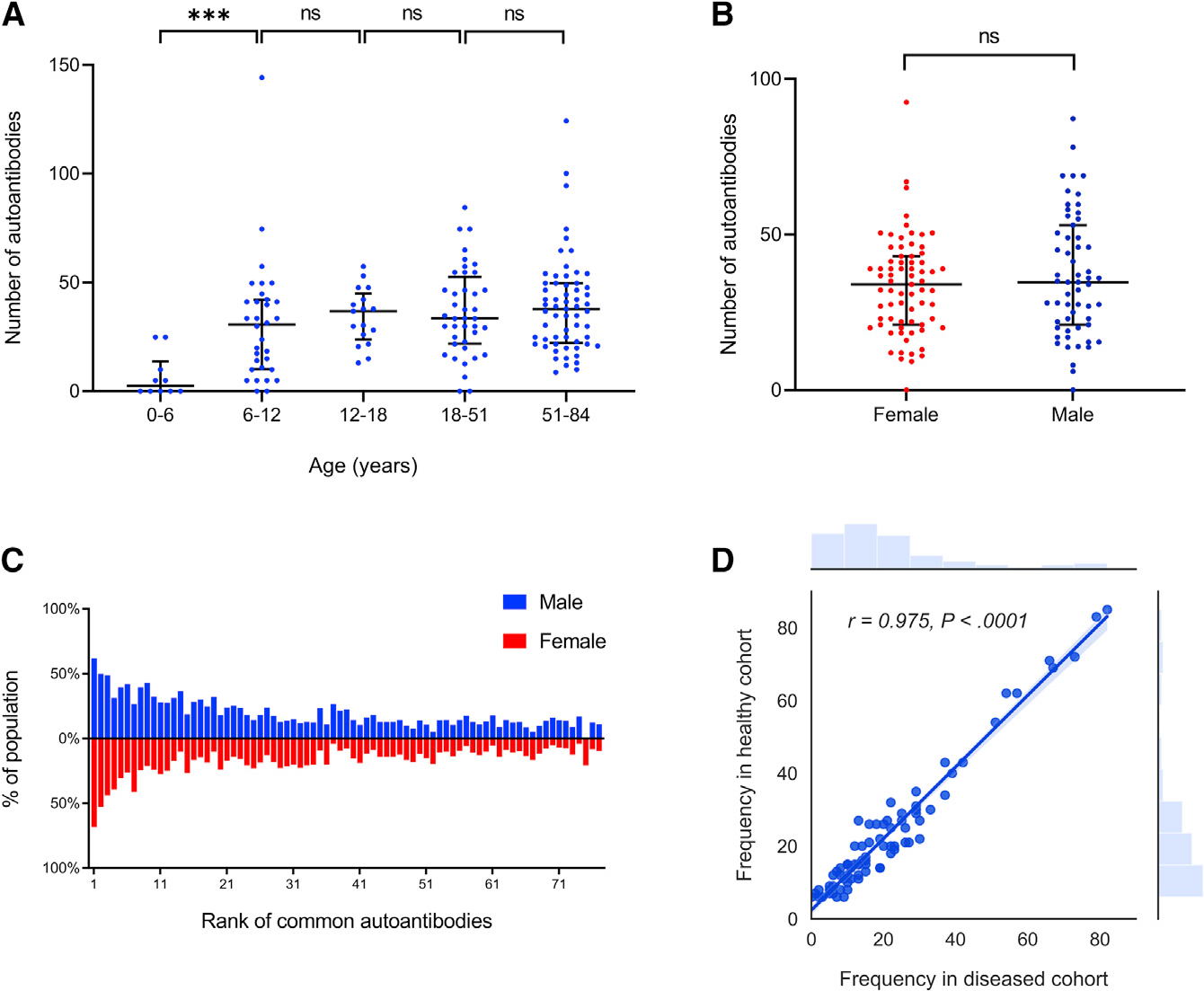
Autoantibody development in healthy subjects (A) All subjects were divided into five age groups based on human development stages. Each blue dot represents the number of autoantibodies found in a healthy subject belonging to that age group. The number of autoantibodies increased significantly over the first two age groups (p < 0.001). The horizontal black bar represents median with interquartile range. (B) Comparison of number of autoantibodies in female and male healthy subjects. A red dot represents the number of autoantibodies found in a single female subject while a blue dot represents the same in a single male subject. There were no significant differences between male and female for the number of autoantibodies (two-sample unpaired t test, p = 0.17). The horizontal black bar represents median with interquartile range. (C) Comparison of weighted prevalence of common autoantibodies in male and female healthy subjects. A blue bar represents the weighted prevalence of a common autoantibody in the male population, while a red bar below the blue one represents the weighted prevalence of the same autoantibody in the female population. Common autoantibodies are ranked from left to right based on their overall prevalence in healthy subjects. Names of the autoantigens and their ranks are listed in the Table S2. No significant difference between male and female for the weighted prevalence was observed (paired t test, p = 0.06). (D) Pearson correlation of common autoantibody frequency in healthy and diseased cohorts (*r* = 0.975). Each dot represents an autoantigen, against which the autoantibody frequency in either cohort is shown.

To examine the time course of autoantibody development, we divided 160 healthy subjects from five studies that included age information (studies I, II, IV, VI, and VII; Table S1) into five age groups based on human development stages. The infant- and early-childhood-age group (0–6 years) had the least number of autoantibodies. The number increased in the middle- and late-childhood-age group (6–12 years) and then plateaued (Figure 1A; p < 0.001). To investigate whether the number or identity of autoantibodies showed a gender bias, we compiled four studies that included both male and female subjects with matched ages (studies I, II, IV, and VII) and compared the counts and identities of the antibodies. The median numbers of autoantibodies for male and female subjects were similar (Figure 1B; p = 0.17). The weighted prevalence of 77 common autoantibodies also had comparable distribution between male and female subjects (Figure 1C; p = 0.06).

We reasoned that if these common autoantibodies observed in the healthy subjects were elicited through common non-pathogenic mechanisms, they should also occur at similar frequencies in their matched disease cohorts. Indeed, the 77 common autoantibodies occurred at similar frequencies in diseased cohorts to those in healthy cohorts (Figure 1D; Pearson correlation coefficient *r* = 0.975).

We wondered if any of these common autoantibodies were related to each other; that is, was there any concordance among them, or were their occurrences independent? We analyzed the common autoantigens pairwise to determine if any occur together in healthy individuals at frequencies greater than chance alone (Figure S2). We found that the majority of them were independent of each other except several pairs: EDG3 and EPCAM (Phi correlation coefficient: 0.83), PML and PSMD2 (Phi correlation coefficient: 0.73), and EPCAM and CSF3 (Phi correlation coefficient: 0.67). Moreover, when we looked at these pairs in the diseased individuals, their concordance was also elevated (Figure S2).

### Sequence similarity with viral proteins

**Figure 2.**
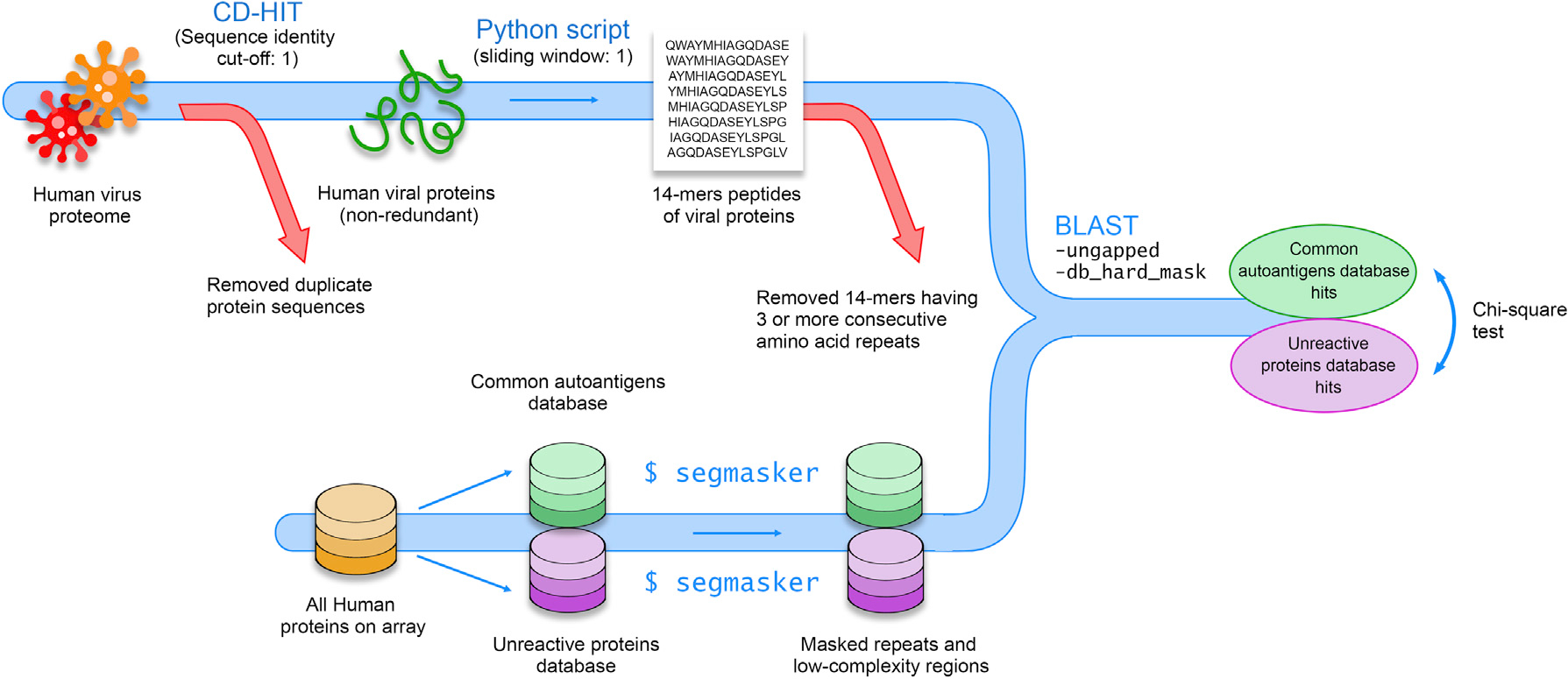
Sequence similarity between common autoantigens and viral proteins Schematic diagram for the discovery of 7 or more ungapped amino-acid matches between common autoantigens and viral proteins.

To understand the extent that common autoantibodies observed in our study resulted from cross-reactivity of antibodies induced by viral infection, we examined the sequence similarities between viral proteins and common autoantigens. As these autoantibodies developed early in age and did not change after adolescence, respiratory and common viruses found in children of the United States were included in the analysis (Table S3). In order to avoid redundancy and false positives, duplicate proteins and consecutive amino-acid repeats were removed from viral proteomes (Figure 2). Similarly, human proteins were masked to avoid repeats and low-complexity regions (homopolymeric runs, short-period repeats, and over representation of one or few residues) as potential hits. Using 7 ungapped amino-acids matches as the threshold, we identified 28 instances of 7 ungapped amino-acid matches and 1 instance of 8 ungapped aminoacid matches with viral proteins that were present in 21 common autoantigens (Table 1). Some of the matches were from the peptides of high-complexity regions like SYFGLRT, LRQEINA, WPEGYQL, and ARCETQN. To assess if these matches were statistically significantly higher than random chance, we calculated the total sequence matches above the threshold for the unreactive proteins (i.e., proteins without any autoantibody response) against the same set of viral proteins. To control for increased chance of a match due to protein length, the results were normalized and expressed as frequency at the amino-acid level. There were 201 amino acids in matched peptides higher than the threshold among 34,070 amino acids of the common autoantigens, while 5,801 amino acids matched higher than the threshold among 2,026,890 amino acids of the unreactive proteins (chi-square test, p < 0.00001).

### Biochemical and structural properties

**Figure 3.**
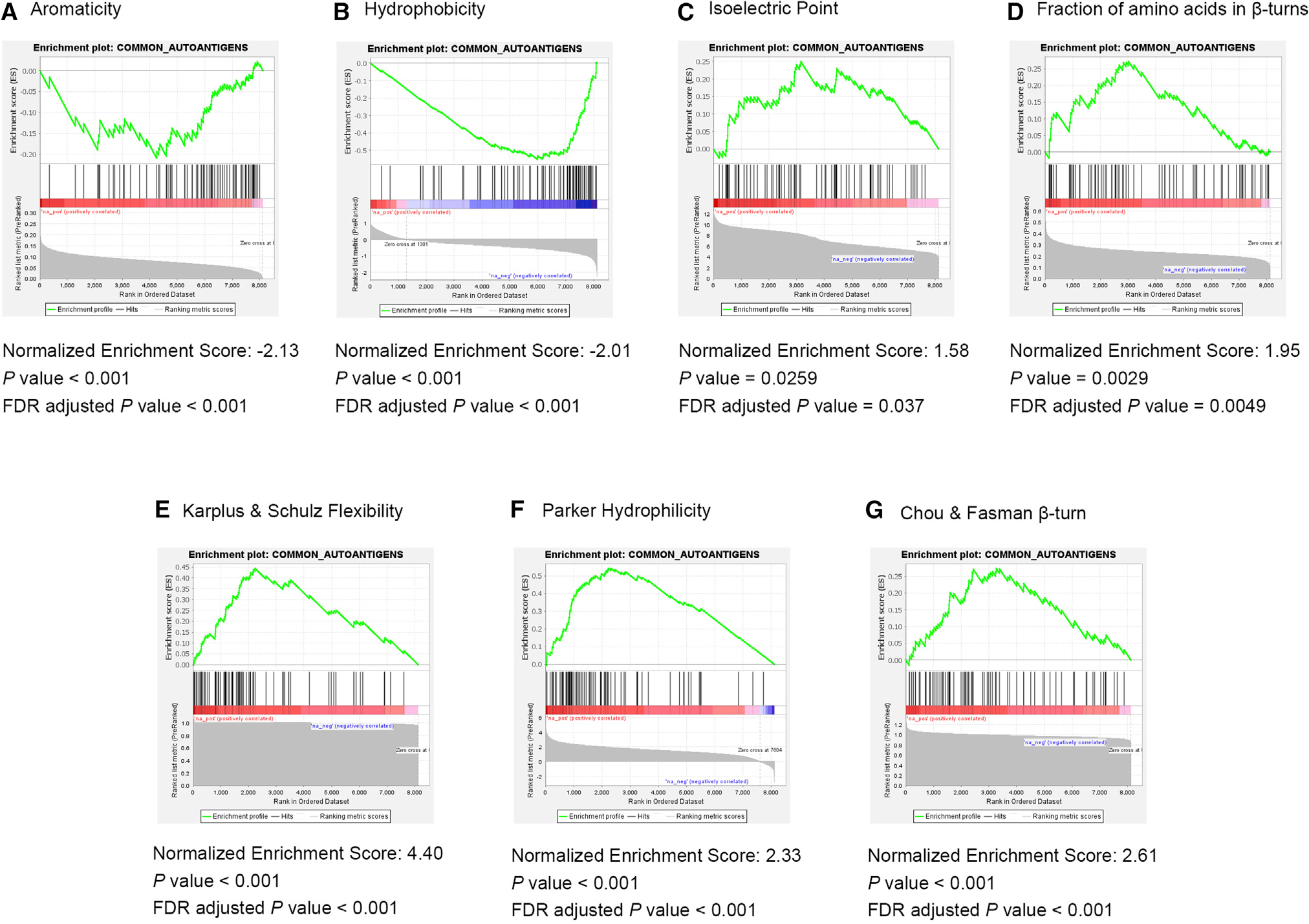
GSEA of common autoantigens for various biochemical and structural properties (A–D) Primary structure enrichment analysis as labeled. (E–G) Antigenicity and secondary structure prediction method enrichment analysis as labeled. The gray colored curve on the graph represents the values of the property sorted in descending order for all the proteins studied. The black vertical lines on the graph show where the common autoantigens appear in the ranked list. The green curve corresponds to the enrichment score, which is calculated by walking down the ranked list, increasing it when a gene is encountered from the gene set and decreasing it when the encountered gene is not from the gene set. The red color gradient is used to represent positive values, while the blue color gradient is used to represent negative values. Concentration of vertical lines on the graph toward a side signifies enrichment, while randomly dispersion of vertical lines on the graph signifies no enrichment.

We asked whether any intrinsic biochemical and structural properties of the target antigens were responsible for common autoantibody production. We examined various properties by comparing our list of common autoantigens with all 8,282 proteins using gene set enrichment analysis (GSEA). The 77 common autoantigens were significantly enriched with proteins having low aromaticity (normalized enrichment score [NES]: −2.13, p < 0.001), low hydrophobicity (NES: −2.01, p < 0.001), high isoelectric point (NES: 1.58, p = 0.018), high fraction of amino acids in beta turns (NES: 1.95, p = 0.04), high Karplus and Schulz flexibility (NES: 4.40, p < 0.001), high Parker hydrophilicity (NES: 2.33, p < 0.001), and high Chou and Fasman beta-turn score (NES: 2.61, p < 0.001) (Figure 3). However, other biochemical properties such as protein length, the fraction of amino acids in beta sheets, and Emini surface accessibility showed no significant enrichment (Figure S3).

### Subcellular localization and tissue expression

**Figure 4.**
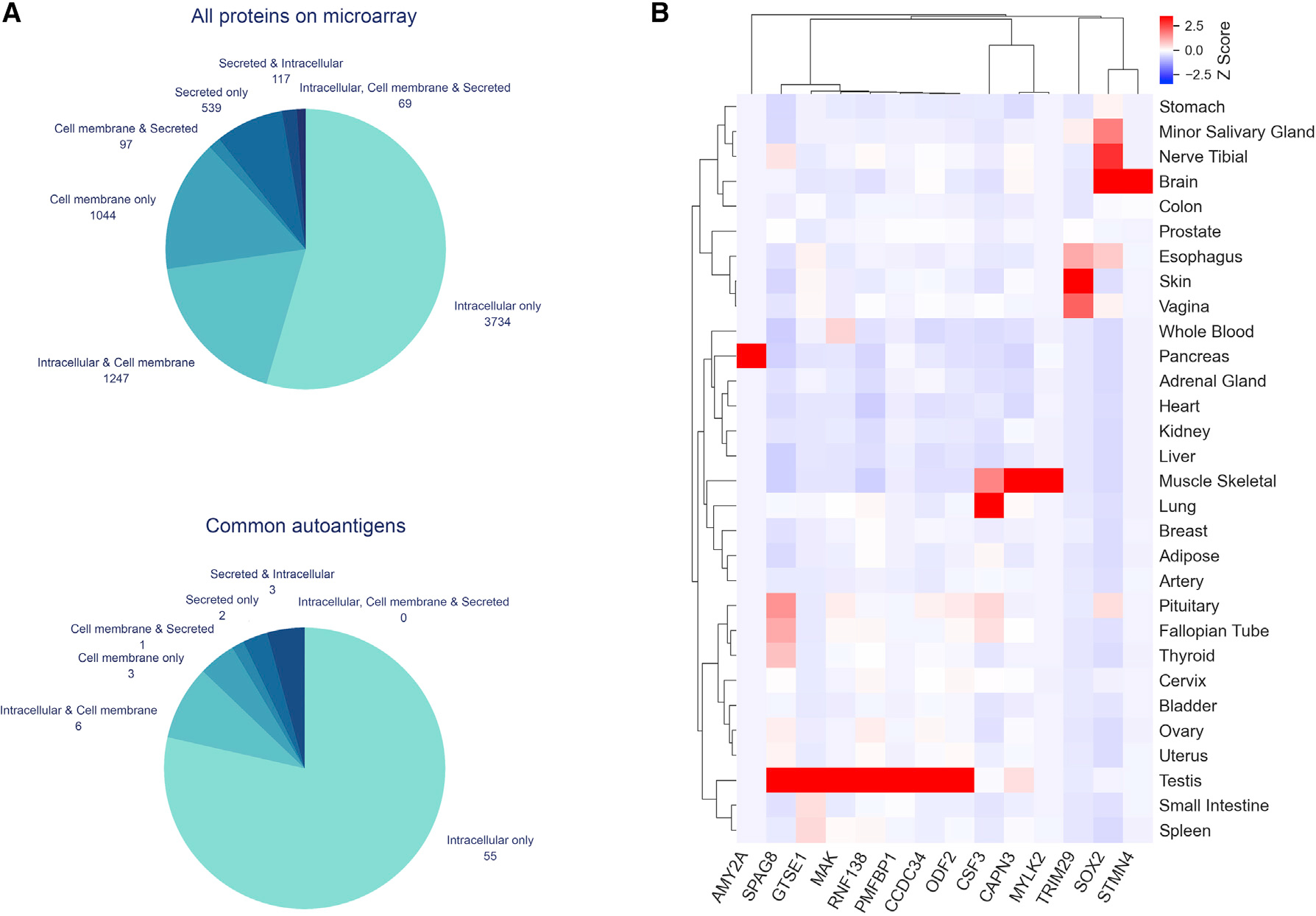
Subcellular localization and tissue expression of common autoantigens (A) Subcellular localization of all proteins and common autoantigens on the microarrays. (B) Expression profiles of organ/tissue-specific common autoantigens. Each row represents an organ as labeled on the right, and each column represents an autoantigen as labeled at the bottom. Gene expression in transcripts per million (TPM) from GTEx dataset was standardized to the *Z*-scores for data visualization. Organs and autoantigens were clustered based on correlation-based average-linkage clustering.

The discovery of common autoantibodies in healthy individuals raised the question about why these antibodies do not lead to autoantibody-mediated pathology. A primary requirement for such pathology is the formation of immune complexes. We examined the subcellular localization of the common autoantigens to see if they were antibody accessible. We divided them into three broad categories: “intracellular,” “cell membrane,” and “secreted” (Table S5). The localization of an autoantigen can belong to one or more of these 3 categories. We found that 55 among 70 common autoantigens were located exclusively at intracellular sites. The percentage of common autoantigens with intracellular-only subcellular localization was significantly higher than that for all the proteins studied on the microarrays (78% versus 54%, p < 0.001) (Figure 4A).

Tissue-specific gene expression can impact autoantigen exposure to circulating autoantibodies and the potential to trigger autoimmune disease. We used the data from GTEx, which is a public resource portal for tissue-specific gene expression in multiple human tissues. In the GTEx dataset, transcripts encoding for 14 common autoantigens were organ/tissue-specific (defined as having log_2_ ((organ expression)/(mean expression in all other organs) > 3) (Figure 4B). Among them, *PMFBP1*, *ODF2*, *RNF138*, and *CCDC34* were predominately expressed in testis, while *STMN4* and *SOX2* were predominantly expressed in the brain. For instance, *PMFBP1* has 29.47 transcripts per million (TPM) in testis, while the mean in other organs is 0.48 TPM. Similarly, *STMN4* has 77.23 TPM in the brain, while the mean in other organs is 0.32 TPM. Other common autoantigens did not show tissue specificity (Figure S4).

## DISCUSSION

Autoantibodies can be broadly divided into two types: (1) pathogenic autoantibodies that contribute to various immune-mediated diseases and (2) common autoantibodies that are found in apparently healthy individuals. While pathogenic autoantibodies can lead to autoimmune diseases, common autoantibodies can bind to a variety of microbial components, thereby providing the first line of defense against infections (Elkon and Casali, 2008). They can also recognize self antigens which help in B cell repertoire development and homeostasis of the immune system. Some of these common autoantibodies occur frequently enough to confound studies intended to find disease-related autoantibodies.

The number of unique IgG autoantibodies in healthy individuals increased with age from infancy to adolescence and then plateaued. This observation suggests that while response to infectious agents (and maybe vaccines) might contribute to autoantibodies through molecular mimicry, this mechanism does not appear to continue to accumulate autoantibodies throughout life. Gender did not appear to play a role in autoantibody production in healthy individuals, which is in agreement with the results reported by Neiman et al. (2019). This stands in contrast to the observation that autoimmune diseases disproportionally affect females compared with males because male-predominant autoimmune disease is associated with acute inflammation, whereas female-predominant autoimmune disease is associated with antibody-mediated pathology (Fairweather et al., 2008). We noted that several common autoantibodies co-occurred frequently. This could occur if the same antibody recognized two different proteins that share a common epitope. Other possibilities include sharing common human leukocyte antigen (HLA) haplotypes or playing similar biological roles that lead to escape from tolerance. It is notable that the targets of several of the co-occurring antibodies play roles in stem cell proliferation and differentiation (EPCAM, EDG3, and CSF3) and two others play roles in DNA-damage repair (PML and PSMD2). The meaning of this is not clear, but it occurred frequently enough (Phi correlation coefficient >0.6) that it is worth further investigation.

Viral proteins with sequences similar to a human protein may initiate cross-reactive antibodies leading to autoimmunity. There are around 20 autoimmune diseases reported in literature where autoantibodies are generated due to cross-reactivity to infectious agent proteins (Cusick et al., 2012). We reasoned that some of the common autoantibodies may be a result from cross-reactivity from anti-viral antibodies, albeit without causing subsequent pathology. The typical length of linear epitope of antibodies ranges from 7 to 9 amino acids, and hence, these specific matches have the potential to elicit cross-reactive antibodies (Buus et al., 2012; Dunn et al., 1999). The fact that these matches occur significantly more frequently between viral proteins and common autoantigens, but less frequently for unreactive proteins on the microarrays, further suggests the role of molecular mimicry in common autoantibody elicitation.

The intrinsic properties of a protein, such as its chemical and structural complexity, can impact its antigenicity (Berzofsky, 1985). Based on our GSEA, we found that common autoantigens tended to favor more basicity, hydrophilicity, and fewer aromatic amino acids. In addition, common autoantigens were also found to be more flexible and have more beta turns. Flexibility is a property that can help the polypeptide chain to bind easily to Ig compared with a stiff polypeptide chain (Berzofsky, 1985). Also, beta turns can be a potential site for antibody binding as the peptide chain reverses its direction at beta turns with the side chain projected outwards (Rose et al., 1985).

Accessibility of autoantigens to circulating autoantibodies is critical to autoimmune disease pathology (Janeway et al., 2001). In systemic autoimmune diseases, a majority of the target antigens are intracellular molecules and therefore not normally accessible to the B cells or antibodies (Suurmond and Diamond, 2015). Only after excessive cell death or ineffective clearance of apoptotic debris do these intracellular autoantigens become available for immune complex formation. In Wegener’s granulomatosis, the autoantigen is an intracellular protease that becomes accessible to the autoantibodies only after an infection triggers translocation of the protease to the surface (Janeway et al., 2001). Similarly, the autoantigen in Goodpasture’s syndrome, normally ensconced in the basal membranes of alveolar capillaries, becomes accessible to the antibodies after an environmental insult to the capillaries, leading to pulmonary hemorrhage (Janeway et al., 2001). A majority of the common autoantigens we identified were located exclusively at intracellular sites, which make them inaccessible to circulating autoantibodies. We also found that some of the common autoantigens are organ/tissue specific and predominately expressed in the testis and brain, which are isolated from the immune system by the blood-testis or blood-brain barriers, respectively. No obvious form of sequestration was identified for the remaining autoantigens, although this cannot be ruled out.

Thousands of studies over the past decade have investigated autoantibodies as potential biomarkers for disease risk assessment, diagnosis, and prognosis (Leslie et al., 2001; Yadav et al., 2019). Given the prevalence we observed for these common autoantibodies in healthy individuals, in some cases exceeding a quarter of all individuals, they will be frequently encountered in such studies and may confound them as false positives. An examination of the AAgAtlas and PubMed revealed that 20 of our 77 common autoantibodies have been reported as disease-related biomarkers (Table S2). Although membership among the common autoantibodies found here does not exclude the possibility that an antibody could not also be disease specific, it would certainly be beneficial for authors to know which autoantibodies commonly occur in healthy individuals (Dervan et al., 2010; Frostegård et al., 2018). It is also now evident that a holistic approach to understanding autoimmunity at the omics level is important in addition to the individual antibody level (Moritz et al., 2020).

### Limitations of the study

Our study used subjects from different studies performed at different times, some with smaller protein subsets, and an overall moderate number of samples. While these factors do not limit the validity of the common autoantibodies found here, they limit the statistical power for finding less prevalent ones. There were more samples from female than male participants. We did not see differences in direct comparison, but we might be lacking the power to find common autoantibodies in males. Overall, the study examined less than half of all human proteins, so examination of the remaining proteins would likely reveal additional common autoantibodies not found here. The potential role of viruses in eliciting common autoantibodies requires more experimental evidence. The use of linear-epitope matching may miss some three-dimensional epitopes. Also, in the future, access to history of viral infection in the healthy subjects would provide a point of comparison with the data and more confidence in potential molecular mimicry.

## STAR★METHODS

### RESOURCE AVAILABILITY

#### Lead contact

Further information about this manuscript and requests for resources will be fulfilled by the Lead Contact, Joshua LaBaer (Joshua.LaBaer@asu.edu).

#### Materials availability

This study did not generate new unique reagents.

#### Data and code availability

All raw binary data have been deposited at Mendeley Data and is publicly available as of the date of publication. DOIs are listed in the key resources table.

Any additional information or code required to reanalyze the data reported in this paper is available from the lead contact upon request.

### METHOD DETAILS

#### Datasets

The healthy subjects included in this study were originally included in 9 different case-control studies (Table S1). These studies were all conducted in our lab; 5 of them were published (Table S1). The serum samples were collected from various parts of the USA and the UK. The goal of the original studies was to discover biomarkers of various cancers and autoimmune diseases by comparing the prevalence of antibodies present in diseased and healthy subjects. The presence of antibody was determined using protein microarrays that displayed thousands of human proteins as potential targets. Serum samples were probed on protein microarrays followed by a secondary antibody with a fluorophore tag specific for human IgG. Microarrays were scanned by a laser scanner. The microarray images from the 9 studies were qualitatively examined to identify protein targets that serum antibodies bound using Array-Pro Analyzer 6.3 (Media Cybernetics) (Bian et al., 2016; Montor et al., 2009). All proteins were not probed by all samples included in our analysis (Figure S1). Several studies focused on female-associated disease and thus only employed samples from females. A table of 8,282 rows of unique proteins and 587 columns of subjects in the case and control groups with binary response data of protein microarrays was created for data and statistical analysis (https://doi.org/10.17632/g57436wy6j.1).

#### Age and gender comparison

To understand the effect of age on autoantibody counts in healthy individuals, studies having both male and female subjects with age information were used (Studies I, II, IV, VI, VII, Table S1). A total of 160 subjects were divided into five age groups based on human development stages. The groups were 0–6 years old (infancy & early childhood), 6 to 12 years old (middle & late childhood), 12 to 18 years old (adolescence), 18 to 51 years old (early adulthood) and 51 to 84 years old (late adulthood). The number of autoantibodies in each subject was plotted using GraphPad Prism by age groups. To understand the effect of gender on autoantibody counts in healthy individuals, studies having both male and female subjects with matched age were used (Studies I, II, IV, VII, Table S1). The subjects were divided into male and female groups. The number of autoantibodies found in each subject was plotted using GraphPad Prism. The weighted prevalence of each autoantibody was calculated for male and female separately. The method of weighted prevalence calculation is described in the “Quantification and statistical analysis” subsection. Prevalence values for the 77 most common autoantibodies were plotted as a population pyramid using GraphPad Prism. A paired t test was performed to determine the significance of the prevalence difference between genders. Pearson correlation of common autoantibodies frequency in diseased and healthy cohorts were plotted using Python seaborn package.

#### Correlation of common autoantibodies

As the presence of common autoantibodies were measured on a binary scale, a phi correlation coefficient (Cramér, 2016) was computed to measure associations between autoantibodies. Specifically, for each pair of antibodies, a phi correlation coefficient was computed for each study, and multiple phi correlation coefficients across different studies were combined into a single phi correlation coefficient using the R meta package. The R “pheatmap” package was then used to produce correlation heatmap plots for both healthy and diseased cohorts (Figure S2). Here, phi correlation coefficient was not defined when one pair of antibodies showed no responses for all the samples, and these undefined phi correlation coefficients were colored as gray on the heatmap plots. Pairs of antibodies having correlation coefficient higher than 0.6 in both cohorts and have correlation in more than one study were validated.

#### Sequence similarity with viral proteins

The proteomes of respiratory and common viruses found in children of the US were downloaded from UniProt as a FASTA file. All the common human viruses were included except sexually transmitted ones as common autoantibodies that develop early in age and then plateau (Table S3). CD-HIT was employed to remove duplicate sequences in the file (sequence identity cut-off: 1) (Huang et al., 2010). The sequences were then segregated into 14-mer peptides using a Python script (sliding window: 1) and consecutive amino acid repeats (3 or more) were removed. The sequences of all the human proteins analyzed on microarrays were retrieved from DNASU (https://dnasu.org) and split into two sequence databases, “common autoantigens” and “unreactive proteins”. The “unreactive proteins” database comprises proteins from the microarrays without any autoantibody responses. Repeats and lowcomplexity regions were masked using BLAST+ (Basic Local Alignment Search Tool, version 2.10.1) package “segmasker” (Galperin, 2003). A protein-protein BLAST was run with the following parameters, “-ungapped, -db_hard_mask 21, -comp_based_stats F, -evalue 10”, between viral 14-mer peptides and “common autoantigens”. Another protein-protein BLAST was run between viral 14-mer peptides and “unreactive proteins” with similar parameters except adjusted “-evalue 593.89” to compensate for the bigger size of unreactive proteins database (Effective search space of “unreactive proteins” and “common autoantigens” databases were 15,970,464 and 268,912, respectively). The total number of amino acids matches higher than the threshold (7 ungapped amino acids match) was calculated for both databases and compared with the total number of amino acids in each database using a chi-square test (Figure 2A).

#### Biochemical and structural properties

Biopython (version 1.75) module Bio.SeqUtils.ProtParam for Python (version 3.7.6) was used to calculate the values of aromaticity, isoelectric point, hydrophobicity, the fraction of amino acids in sheets and turns for each protein (Table S4). Secondary structure and antigenicity prediction methods from Immune Epitope Database (IEDB) were also used. Command-line tools from IEDB analysis resource (http://tools.iedb.org/bcell/download/) were employed to calculate the values of Chou & Fasman beta-turn, Emini surface accessibility, Karplus & Schulz flexibility, and Parker hydrophilicity across the proteins, which were then averaged for each protein. The computed biochemical property values were used for the enrichment analysis on the identified common autoantigens using Gene Set Enrichment Analysis (GSEA) “GSEAPreranked” package (version 4.2) (Subramanian et al., 2005).

#### Subcellular localization and tissue expression

All 8,282 proteins were used to query the UniProt database for subcellular localization (downloaded in December 2020), among which 6,875 proteins had subcellular localization data available in the database (Table S5). Some of the proteins were found simultaneously in more than one location, and hence, seven groups were created to segregate the proteins based on their subcellular localization profiles. Proteins that were found only in one subcellular location were put into “intracellular only”, “cell membrane only” and “secreted only” groups. Proteins that were found in two subcellular locations were put into “intracellular & cell membrane”, “cell membrane & secreted” and “secreted & intracellular” groups. Proteins that were found simultaneously inside the cell, in the cell membrane, and outside the cell were put into “intracellular, cell membrane and secreted” group. p value was calculated to assess the statistical significance of difference in fractions of “intracellular only” proteins for all proteins on the microarrays and for common autoantigens using the proportion test.

All 8,282 proteins were mapped to the Ensembl IDs using “BiomaRt” package available for R (version 3.5.0). The Ensembl IDs were used to identify the protein of interest in the Genotype-Tissue Expression (GTEx, version 8) dataset. The gene expression levels in 52 human tissue types, measured in transcripts per million (TPM), were downloaded from GTEx. Expression values for tissue types belonging to the same organ were averaged. Differentially expressed genes for each organ/tissue were identified using edgeR package for R (version 3.6.2) with a cutoff of Log_2_ (fold change) > 3 to determine organ/tissue specificity, where the fold change for each gene was calculated by dividing the TPM value in a particular organ/tissue by the mean TPM values in all other organs/tissues. The log2-scaled fold changes across the organs/tissues for each gene were standardized to the Z scores for data visualization. The *Z* score profiles were displayed in a heatmap with correlation-based average-linkage clustering by using the Python seaborn package.

### QUANTIFICATION AND STATISTICAL ANALYSIS

#### Weighted prevalence

Due to the heterogeneous number of proteins and subjects being analyzed in each study, we computed the weight for the *j* th antibody as, ${\hat{p}}_{j}=\sum_{i=1}^{k}w_{ij}p_{ij}/\sum_{i=1}^{k}w_{ij}$ where *p*_*ij*_ = *x*_*ij*_*/n*_*ij*_ is the prevalence,*x*_*ij*_ is the total number of positive signals found for the *j*th antibody in the study ,*i* and *n*_*ij*_ is the number of samples for the *j* th antibody in the study ,*i* and *k* is the number of studies. Here, $w_{ij}={\left(v_{ij}+\tau_{j}^{2}\right)}^{-1}$ is the inverse variance-weighting which accounts for the heterogeneous effects between studies (Borenstein et al., 2010), where *v*_*ij*_ = *n*_*ij*_/(*p*_*ij*_(1 − *p*_*ij*_)), $\tau_{j}^{2}=\left(Q_{j}-k+1\right)/U_{j}$ if *Q*_*j*_ *> k* − 1 or *τ*_*j*_^2^ = 0 otherwise, $Q_{j}=\sum_{i=1}^{k}v_{ij}{\left(p_{ij}-p_{j}\right)}^{2}$, $U_{j}=(k-1)({\bar{v}}_{j}-s_{j}^{2}/\left(kv_{j}\right))$, $p_{j}=\sum_{i=1}^{k}v_{ij}p_{ij}\sum_{i=1}^{k}v_{ij}$, $s_{j}^{2}=\left(\sum_{i=1}^{k}v_{ij}^{2}-k{\bar{v}}_{j}^{2}\right)/(k-1)$, and ${\bar{v}}_{j}=\sum_{i=1}^{k}v_{ij}/k$. The same analysis was performed to calculate gender-specific weighted prevalence by splitting the dataset into male and female subsets.

#### Age and gender comparison

The significance of increase in the autoantibody counts among the five age groups was calculated using the Welch’s t test while the significance of difference in autoantibody counts between the male and female groups was calculated using a two-sample unpaired t test.

#### Biochemical and structural properties

The “GSEAPreranked” package available in GSEA software returned p values of “0.0” when the number of permutations was set to 1,000, as it cannot calculate very small p values. Another R package named “fgsea” was used to calculate the very small p values with number of permutations set to 10,000 for more accurate calculation. To adjust multiple comparisons, we computed false discovery rate (FDR) adjusted p value using the “p.adjust” function in the R stats package.

## Supplementary Material

1

2

3

## Figures and Tables

**Table 1.** Sequence similarity of common autoantigens and viral proteins

S. no.	Autoantigen	Viral UniProt ID	Sequence similarity	Organism

1	ADNP2	P16812	LPVPPGG	human herpesvirus 5
		H9C1C1	SYFGLRT	human rotavirus C
2	AHCY	F8WQQ3	GKLNVKL	human adenovirus 41
3	AMY2A	P16766	SAGTSST	human herpesvirus 5
4	APEX2	M1JRT8	NRSGYSG	influenza A virus
		P09289	ALLAAGS	human herpesvirus 3
5	C9orf78	P16764	EDCLYEL	human herpesvirus 5
6	CTTNBP2NL	P52529	EQLRAKL	human herpesvirus 6A
		C4AL53	AKLNREE	influenza A virus
		Q6SW92	SSNTVVA	human herpesvirus 5
7	FLJ36888	P52355	TIKRTLV	human herpesvirus 7
8	KAZ	O09800	ARCETQN	human herpesvirus 1
9	MAK	P16793	GTSEVDE	human herpesvirus 5
		Q01350	WPEGYQL	human herpesvirus 6A
		Q69513	KSDSELS	human herpesvirus 7
10	MAPK13	Q8QT31	VIGLLDV	human parainfluenza virus 1
11	MTUS2	P09284	IDQNTVV	human herpesvirus 3
		A0A0D5Z8N5	SPIKLSP	rotavirus B
12	MYLK2	Q6SWD0	AEEGKNI	human herpesvirus 5
13	PAK1	P24433	SVIEPLP	human herpesvirus 6A
14	PAK7	P16739	ATAQELL	human herpesvirus 5
15	PELI1	Q9QJ30	LRQEINA	human herpesvirus 6B
16	PML	A0MK42	TLGAVVP	human adenovirus 52
17	RABGEF1	I1V183	SPRKQEAE	human adenovirus 7
18	SECISBP2	D3JIS2	ELTVAAR	human adenovirus 18
19	TAF1D	P09252	DATHLED	human herpesvirus 3
20	TRAP1	P0C723	ALIRKLR	Epstein-Barr virus
		P10200	AQLGPRR	human herpesvirus 1
21	ZNF688	Q1HVD1	GAQPPAP	Epstein-Barr virus

Common autoantigens with 7 or more ungapped amino acids that match with viral proteins are reported along with virus name and the corresponding sequences.

**KEY RESOURCES TABLE:** 

REAGENT or RESOURCE	SOURCE	IDENTIFIER

Deposited data		

Autoantibody reactivity raw binary data	Mendeley Data	10.17632/g57436wy6j.1

Software and algorithms		

Array-Pro Analyzer 6.3	Media Cybernetics	N/A
Prism 9	GraphPad	https://graphpad.com/
R 3.5	R Foundation	https://r-project.org/
RStudio	RStudio PBC	https://rstudio.com/
Python 3.7.6	Python Software Foundation	https://python.org/
Spyder 4.1.4	Spyder project contributors	https://spyder-ide.org/
Anaconda 1.9.12	Anaconda Inc.	https://anaconda.com/
CD-HIT	Weizhong Li’s group	http://cd-hit.org/
MobaXterm 20.1	Mobatek	https://mobaxterm.mobatek.net/
BLAST 2.10.1	National Center for Biotechnology Information	https://blast.ncbi.nlm.nih.gov/
IEDB	National Institute of Allergy and Infectious Disease	http://iedb.org/
GSEA 4.2	UC San Diego and Broad Institute	https://gsea-msigdb.org/
DAVID 6.8	Laboratory of Human Retrovirology and Immunoinformatics	https://david.ncifcrf.gov/
GTEx 8	GTEx Consortium	https://gtexportal.org/
UniProt	UniProt Consortium	https://uniprot.org/
DNASU	Arizona State University	https://dnasu.org/
Photoshop	Adobe Inc.	https://www.adobe.com/products/photoshop.html

## Highlights

- Meta-analysis reveals 77 common autoantibodies found in healthy individuals
- Autoantibodies in healthy individuals increase with age and then plateau at adolescence
- Sequence similarity with viral proteins likely elicits a subset of these antibodies
- Several intrinsic properties of common autoantigens are enriched
