# Supplementary material for: Serum autoantibodyome reveals that healthy individuals share common autoantibodies
Source: Cell Rep. Author manuscript; Available in PMC 2022 Jun 23. (PMC9221390; doi:10.1016/j.celrep.2022.110873)
Supplement: 1 [file NIHMS1812246-supplement-1.pdf]

Cell Reports, Volume *39*

Supplemental information

Serum autoantibodyome reveals that healthy individuals share common autoantibodies

Mahasish Shome, Yunro Chung, Ramani Chavan, Jin G. Park, Ji Qiu, and Joshua LaBaer

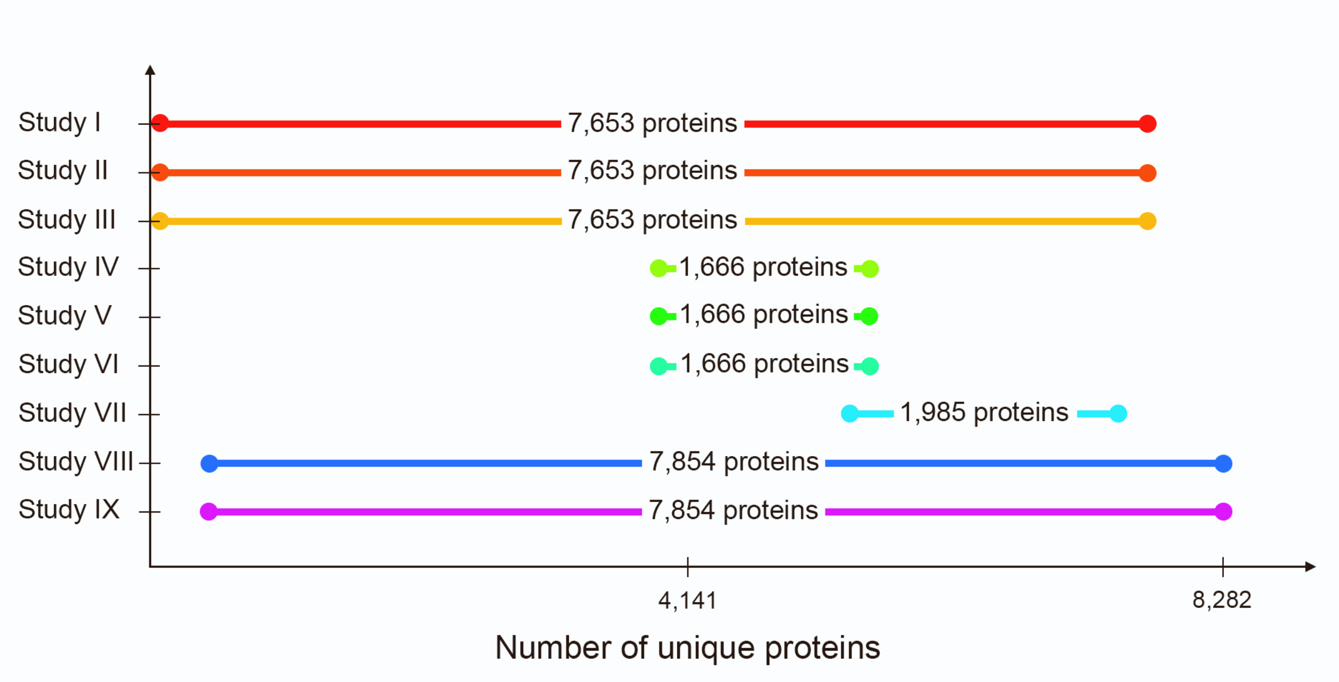

Figure S1. Proteins investigated by the 9 studies used for the meta-analysis, Related to STAR Methods. All 8282 unique human proteins are represented on the x-axis and the proteins analyzed in each study is shown as a line overlapping with the x-axis corresponding to the labels on the y-axis. There were 123 proteins studied by all studies and 7,242 proteins by studies I, II, III, VIII and IX.

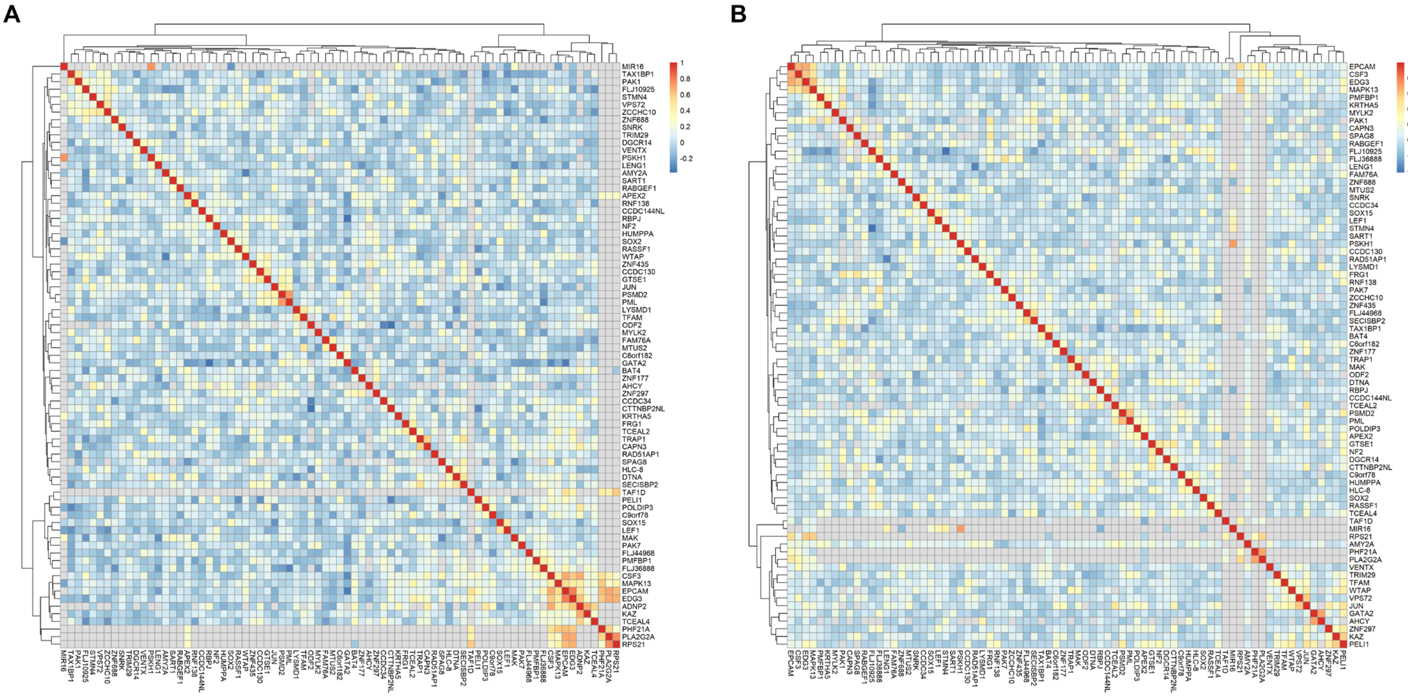

C

Alias1	Alias2	Phi correlation coefficient (healthy)	Phi correlation coefficient (diseased)	Number of studies having correlation	*P* value
**EDG3**	**EPCAM**	**0.83**	**0.67**	**6**	**<0.001**
RPS21	PLA2G2A	0.76	0.53	2	<0.001
**PML**	**PSMD2**	**0.73**	**0.63**	**3**	**<0.001**
PLA2G2A	EPCAM	0.69	0.44	2	<0.001
PLA2G2A	EDG3	0.68	0.19	2	<0.001
**CSF3**	**EPCAM**	**0.67**	**0.65**	**6**	**<0.001**
RPS21	EDG3	0.66	0.58	3	<0.001
PHF21A	EPCAM	0.63	0.31	2	<0.001
RPS21	EPCAM	0.63	0.52	3	<0.001

Figure S2 Correlation of co-occurrence of common autoantibodies in healthy and diseased cohorts, Related to STAR Methods. Phi correlation coefficient was calculated for each pair of autoantibodies and shown as a heatmap for (A) healthy cohort and (B) diseased cohort. The grey color on the heatmaps represent pairs of autoantibodies whose phi correlation coefficient was not defined. (C) Pairs that have correlation coefficient higher than 0.6 in healthy cohort and that have correlation in at least two studies were shown with their corresponding value in diseased cohort. Pairs that have correlation coefficient higher than 0.6 in both the cohorts are shown in bold. *P* value was calculated using a normal approximation from the “metacor” function in the R meta package.

A Protein Length

B Fraction of amino acids in β-Sheets

C Emini Surface Accessibility

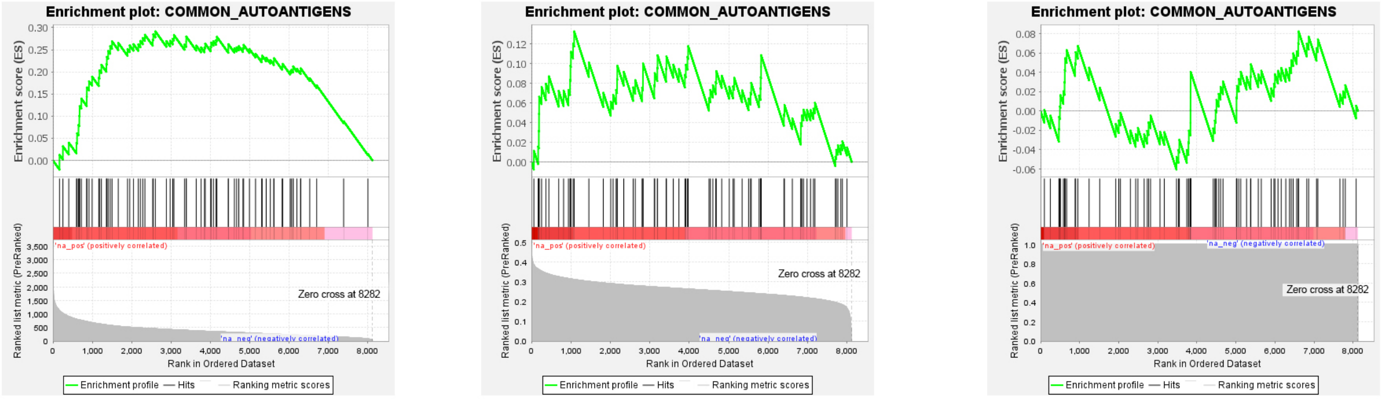

A

Normalized Enrichment Score: 1.07

P value = 0.3525

FDR adjusted *P* value = 0.4407

B

Normalized Enrichment Score: 1.02

P value = 0.4335

FDR adjusted *P* value = 0.4817

C

Normalized Enrichment Score: 0.83

P value = 0.6728

FDR adjusted *P* value = 0.6728

Figure S3 GSEA of common autoantigens for various biochemical and structural properties, Related to Figure 3. (A) protein length; (B) fraction of amino acids in beta-sheets; and (C) surface accessibility prediction. These properties did not lead to significant enrichment. The grey colored curve on the graph represents the values of the property sorted in descending order for all the proteins studied. The black vertical lines on the graph show where the common autoantigens appear in the ranked list. The green curve corresponds to the enrichment score, which is calculated by walking down the ranked list, increasing it when a gene is encountered from the gene set and decreasing it when the encountered gene is not from the gene set. The red color gradient is used to represent positive values while the blue color gradient is used to represent negative values. Concentration of vertical lines on the graph towards a side signifies enrichment while randomly dispersion of vertical lines on the graph signifies no enrichment.

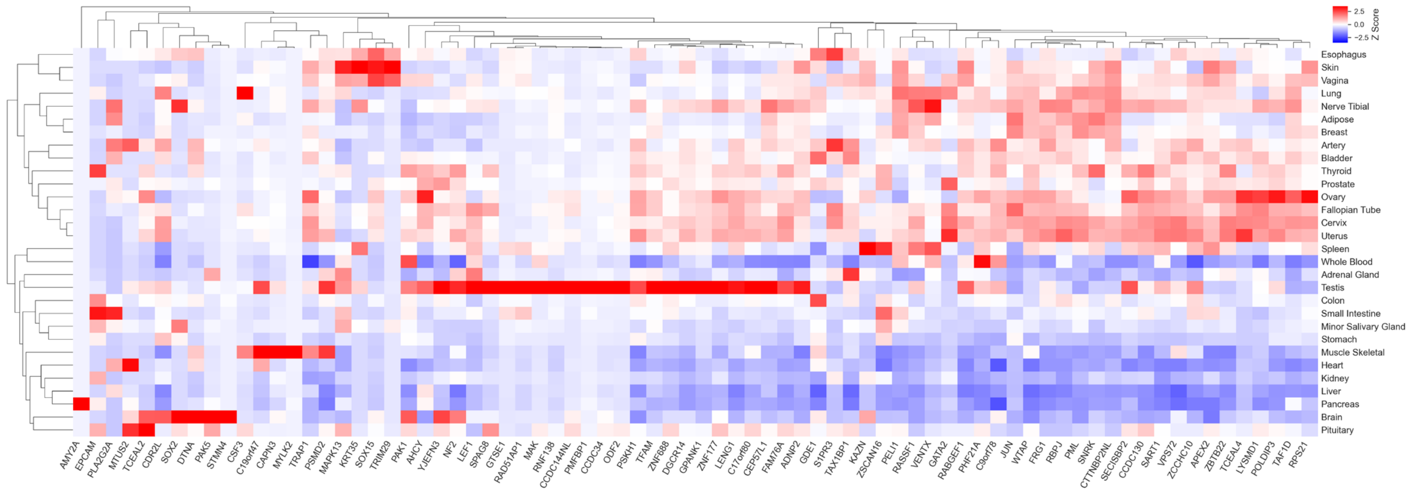

Figure S4 Expression profiles of common autoantigens in different organs, Related to Figure 4B. Each row represents an organ as labelled on the right and each column represents an autoantigen as labelled at the bottom. Gene expression in transcripts per million (TPM) from GTEx dataset was standardized to the Z scores for data visualization. Organs and autoantigens were clustered based on correlation-based average-linkage clustering.

Table S1. Study summary with demographic information, Related to STAR Methods. Each study was performed independently with age and gender matched case-control samples. Some studies focused on female-specific diseases without samples from male subjects.

Study	Healthy subjects	Diseased subjects	Healthy Male	Healthy Female	Age (median)	No. of proteins studied	Reference
I	40	40	14	26	13	7,653	(Bian et al., 2016)
II	40	40	19	21	71.5	7,653	(J. Wang et al., 2016)
III	45	45	0	45	--	7,653	(Wang et al., 2015a)
IV	40	40	21	19	51	1,666	
V	10	20	--	--	--	1,666	(Wang et al., 2017)
VI	30	50	8	22	7.11	1,666	
VII	10	21	5	5	51.5	1,985	
VIII	30	30	0	30	--	7,854	(Katchman et al., 2017)
IX	27	29	0	27	50	7,854	
Total	272	315				8,282 (unique)	

"--" represents "data not available".

Table S2. Weighted prevalence of common autoantibodies in healthy and diseased cohorts, Related to Figure 1. Availability of the autoantibodies in AAgAtlas / PubMed literature were reported as yes [Y] or no [N].

Gene name	No. of reactivity (Healthy)	Total samples (Healthy)	Weighted prevalence (Healthy)	No. of reactivity (Diseased)	Total samples (Diseased)	Weighted prevalence (Diseased)	Found in AAgAtlas / PubMed
STMN4	83	192	0.47	79	205	0.37	Y
ODF2	85	178	0.42	82	182	0.38	Y
RBPJ	69	178	0.37	67	182	0.33	Y
AMY2A	71	167	0.34	66	169	0.33	Y
EPCAM	72	205	0.31	73	235	0.28	Y
ZNF688	54	192	0.29	51	205	0.26	Y
CSF3	62	259	0.25	54	293	0.19	Y
RAD51AP1	30	178	0.23	33	182	0.24	N
PSKH1	15	140	0.23	12	140	0.18	N
LENG1	43	192	0.22	42	205	0.18	N
S1PR3	62	269	0.21	57	314	0.17	Y
LYSMD1	30	178	0.21	29	182	0.17	N
FAM76A	35	178	0.20	29	182	0.20	N
CDR2L	29	192	0.20	25	205	0.12	Y
CCDC130	27	142	0.20	21	143	0.16	N
SOX15	43	192	0.20	37	205	0.14	N
PLA2G2A	15	134	0.19	10	168	0.13	N
TAF1D	29	133	0.19	29	167	0.17	N
CEP57L1	25	178	0.19	26	182	0.16	N
RASSF1	26	140	0.18	20	140	0.14	Y
PHF21A	12	147	0.18	13	190	0.16	N
POLDIP3	40	182	0.18	39	183	0.20	N
ESS2	34	192	0.17	37	205	0.15	N
C17orf80	32	182	0.17	22	183	0.12	N
C9orf78	15	85	0.17	10	85	0.10	N
MTUS2	16	178	0.16	15	182	0.15	N
GDE1	9	140	0.16	6	140	0.12	N
PMFBP1	25	192	0.16	22	205	0.17	N
KAZN	22	125	0.16	19	125	0.14	N
SPAG8	14	182	0.16	8	183	0.10	Y
CCDC144NL	15	125	0.15	15	125	0.09	N
SNRK	21	140	0.15	26	140	0.18	N
CCDC34	26	182	0.15	18	183	0.11	N
MAP11	11	182	0.14	9	183	0.14	N
TRAP1	8	139	0.14	5	143	0.09	Y
SART1	21	182	0.14	16	183	0.09	Y
CTTNBP2NL	20	125	0.14	22	125	0.13	N
KRT35	27	182	0.13	25	183	0.14	N
WTAP	8	178	0.13	10	182	0.10	N
TCEAL4	21	182	0.13	27	183	0.16	N

C19orf47	8	182	0.13	2	183	0.04	N
GATA2	8	192	0.13	8	205	0.03	Y
ZNF177	13	192	0.12	15	205	0.08	N
PSMD2	18	140	0.12	22	140	0.16	N
PML	19	140	0.12	23	140	0.16	Y
SOX2	27	192	0.12	30	205	0.12	Y
MAK	20	140	0.12	14	140	0.09	N
FRG1	22	178	0.12	30	182	0.18	N
ZSCAN16	20	192	0.12	23	205	0.11	N
TRIM29	20	192	0.12	20	205	0.10	N
PAK5	20	192	0.12	12	205	0.08	N
PELI1	7	178	0.11	1	182	0.02	Y
GTSE1	14	179	0.11	19	183	0.13	N
MAPK13	31	259	0.11	29	293	0.11	N
APEX2	27	259	0.11	13	293	0.04	N
VPS72	11	192	0.11	13	205	0.05	N
MYLK2	12	140	0.11	6	140	0.04	N
TAX1BP1	6	140	0.11	7	140	0.12	Y
LEF1	26	192	0.11	16	205	0.06	N
AHCY	6	192	0.10	2	205	0.04	N
ADNP2	6	125	0.10	0	125	0	N
RPS21	12	147	0.10	8	190	0.13	N
TCEAL2	7	192	0.10	6	205	0.03	N
RABGEF1	16	125	0.10	14	125	0.11	N
TFAM	10	125	0.10	10	125	0.10	N
GPANK1	14	259	0.10	19	293	0.05	N
CAPN3	12	178	0.10	10	182	0.10	N
DTNA	6	178	0.10	3	182	0.05	N
ZCCHC10	6	178	0.10	9	182	0.07	N
VENTX	15	125	0.10	13	125	0.09	N
NF2	14	179	0.10	11	183	0.06	N
YJEFN3	15	182	0.10	13	183	0.07	N
SECISBP2	17	182	0.10	15	183	0.09	N
ZBTB22	13	140	0.10	7	140	0.06	N
RNF138	11	125	0.10	11	125	0.06	N
JUN	7	272	0.10	5	315	0.05	Y
PAK1	9	140	0.10	5	140	0.10	Y

Table S3. Viral strains and number of viral proteins used for sequence similarity analysis, Related to Figure 2 and Table 1. Following strains of respiratory and common viruses found in children of the US were used for the analysis. The reference proteomes from UniProt were included for each virus.

Organism	Strains	Number of proteins
Influenza A virus	(A/Alaska/105/2015(H3N2)), (A/Boston/151/2009(H1N1)), (A/Boston/DOA29/2011(H3N2)), (A/Boston/YGA_01042/2012(H3N2)), (A/California/47/2016(H3N2)), (A/California/VRDL67/2009(H1N1)), (A/California/VRDL364/2009(mixed)), (A/Hawaii/67/2014(H1N1)), (A/Hawaii/74/2015(H3N2)), (A/Houston/JMM_42/2012(H3N2)), (A/Kentucky/16/2015(H1N1)), (A/Louisiana/13/2014(H3N2)), (A/New York/169/2000(H3N2)), (A/New York/441/2001(H1N1)), (A/New York/1144/2008(H3N2)), (A/New York/3052/2009(mixed)), (A/New York/WC-LVD-14-057/2014(H1N1)), (A/Oregon/29/2015(H1N1)), (A/South Carolina/09/2009(H1N1)), (A/Tennessee/F2019A/2011(H3N2)), (A/Utah/06/2016(H1N1)), (A/Virginia/43/2016(H3N2)), (A/Puerto Rico/8/1934 H1N1), (A/South Carolina/1/1918 H1N1)), (A/WS/1933 H1N1)), (swl A/California/04/2009 H1N1)	218
Influenza B virus	(B/Florida/66/2015), (B/Florida/78/2015), (B/Texas/14/1991), (B/Utah/15/2015), (B/Florida/78/2015), (B/Utah/31/2016), (B/Lee/1940)	69
Influenza C virus	(C/Ann Arbor/1/1950)	8
Herpes simplex virus 1	(strain 17) (HHV-1)	73
Varicella-zoster virus	(strain Dumas) (HHV-3), (strain Oka vaccine) (HHV-3)	140
Epstein-Barr virus	(strain AG876) (HHV-4), (strain B95-8) (HHV-4), (strain GD1) (HHV-4)	187
Human cytomegalovirus	(strain AD169) (HHV-5), (strain Merlin) (HHV-5)	525
Human B lymphotropic virus	(strain Uganda-1102) (HHV-6 variant A), (strain Z29) (HHV-6 variant B)	205
Human T lymphotropic virus	(strain JI) (HHV-7)	102
Human rhinovirus A	(strain 41467-Gallo) (HRV-89)	1
Human rotavirus A, B, C, G9P [8]		45
Human adenovirus 21, 21a, 26, 52, 55, 56, A, B, C, D, D, E, F	(HAdV-18), (HAdV-7), (HAdV-1), (HAdV-2), (HAdV-5), (HAdV-17), (HAdV-4), (HAdV-41)	599
Human parainfluenza virus 1, 2, 4a	(strain Washington/1964)	24
Human respirovirus 1, 3		10
Respiratory syncytial virus A, B	(strain A2), (strain S-2) (HRSV-S2), (strain B1)	44
Norwalk virus	(strain GI/Human/United States/Norwalk/1968)	3
Human Enterovirus	(strain USA/BrCr/1970) (EV71), (EV68) (EV-68), Human parechovirus 2 (strain Williamson) (HPeV-2), Coxsackievirus A16, Coxsackievirus B2 (strain Ohio-1), Coxsackievirus B3 (strain Nancy), Coxsackievirus B4 (strain JVB/Benschoten/New York/51)	11
Human metapneumovirus	(strain CAN97-83) (HMPV)	9
**Total**		**2,273**
